# A novel BH3-mimetic, AZD0466, targeting BCL-XL and BCL-2 is effective in pre-clinical models of malignant pleural mesothelioma

**DOI:** 10.1038/s41420-021-00505-0

**Published:** 2021-05-28

**Authors:** Surein Arulananda, Megan O’Brien, Marco Evangelista, Laura J. Jenkins, Ashleigh R. Poh, Marzena Walkiewicz, Trishe Leong, John M. Mariadason, Jonathan Cebon, Srividya B. Balachander, Justin R. Cidado, Erinna F. Lee, Thomas John, Walter D. Fairlie

**Affiliations:** 1 Olivia Newton-John Cancer Research Institute, Heidelberg, VIC Australia; 2 School of Cancer Medicine, La Trobe University, Bundoora, VIC Australia; 3 Department of Medical Oncology, Austin Health, Heidelberg, VIC Australia; 4 Department of Clinical Pathology, University of Melbourne, Melbourne, VIC Australia; 5 Department of Pathology, Austin Health, Heidelberg, VIC Australia; 6 Bioscience, Oncology R&D, AstraZeneca, Boston, MA USA; 7 Department of Biochemistry and Genetics, La Trobe Institute for Molecular Science, La Trobe University, Bundoora, VIC Australia; 8 Present Address: Department of Pathology, St Vincent’s Hospital Melbourne, Melbourne, VIC Australia; 9 Present Address: Peter MacCallum Cancer Centre, Melbourne, VIC Australia

**Keywords:** Apoptosis, Mesothelioma

## Abstract

Malignant pleural mesothelioma (MPM) is an aggressive cancer with treatment limited to Cisplatin and Pemetrexed chemotherapy. Recently, we showed that drugs targeting the BCL-2-regulated apoptosis pathway could kill MPM cell lines in vitro, and control tumor growth in vivo. These studies showed BCL-XL was the dominant pro-survival BCL-2 family member correlating with its high-level expression in cells and patient tumor samples. In this study we show another inhibitor, AZD4320 that targets BCL-XL (and BCL-2), can also potently kill MPM tumor cells in vitro (EC_50_ values in the 200 nM range) and this effect is enhanced by co-inhibition of MCL-1 using AZD5991. Moreover, we show that a novel nanoparticle, AZD0466, where AZD4320 is chemically conjugated to a PEGylated poly-lysine dendrimer, was as effective as standard-of-care chemotherapy, Cisplatin, at inhibiting tumor growth in mouse xenograft studies, and this effect was enhanced when both drugs were combined. Critically, the degree of thrombocytopenia, an on-target toxicity associated with BCL-XL inhibition, was significantly reduced throughout the treatment period compared to other BCL-XL-targeting BH3-mimetics. These pre-clinical findings provide a rationale for the future clinical evaluation for novel BH3-mimetic formulations in MPM, and indeed, other solid tumor types dependent on BCL-XL.

## Introduction

Malignant pleural mesothelioma (MPM) is a rare and aggressive cancer caused by exposure to asbestos^1^. Overall survival rates for MPM (typically around 12 months) are amongst the lowest of all cancers^2^, due in part, to the limited treatment options available for these patients. For over a decade the mainstay therapy for MPM has been Cisplatin plus Pemetrexed combination chemotherapy^3^, although the recent MAPS study showed a modest improvement in overall survival with the addition of Bevacizumab, an anti-angiogenic agent, to this regimen^4^. Trials with immunotherapy have demonstrated some benefit predominantly in biphasic and sarcomatoid MPM subtypes^5,6^, but therapies that have attempted to target some of the pathways governed by genetic defects associated with MPM have largely been disappointing^7^.

In an attempt to provide a new avenue for MPM treatment, we and others^8–10^ have recently investigated a more generic approach of targeting the BCL-2 pro-survival proteins (i.e., BCL-2, BCL-XL, BCL-W, MCL-1 and BFL-1) that frequently have deregulated expression in cancer cells, leading to increased tumor cell survival and treatment resistance^11^. This has been made possible due to the availability of a new class of drugs, the “BH3-mimetics”, that directly engage these proteins with high affinity, much like their natural pro-apoptotic ligands, the BH3-only proteins^12^. Compounds have now been developed that are either directed towards multiple pro-survival proteins (e.g. Navitoclax which binds BCL-2, BCL-XL and BCL-W, and AZD4320 which binds BCL-2 and BCL-XL)^13,14^, or which are more specific; Venetoclax against BCL-2^15^, A-1331852 against BCL-XL^16^, A-1210477, S63845 and AZD5991 against MCL-1^17–19^, and others^12^. Our studies showed that MPM cell lines can be potently killed with BH3-mimetics targeting BCL-XL, though this activity is enhanced by co-treatment with an MCL-1 inhibitor^9^. Notably, BCL-XL and MCL-1 were also shown to be the dominant BCL-2 pro-survival proteins expressed in MPM patient samples^9^.

Venetoclax is now being successfully used in the clinic for treatment of chronic lymphocytic leukemia and is being investigated in other hematological malignancies^20–24^, as well as breast cancer where its combination with Tamoxifen appears promising^25^. However, outcomes with BH3-mimetics in other solid cancers have generally not been as positive to-date. For example, despite promising pre-clinical data, little clinical benefit was observed with Navitoclax in a phase II trial in small-cell lung cancer. A major limitation associated with these drugs is the induction of thrombocytopenia, an on-target, dose-limiting, toxicity associated with potent inhibition of BCL-XL^26^ which is required for platelet survival. Nevertheless, there are currently several trials on-going with Navitoclax in other cancers.

Unlike Navitoclax, the potent dual BCL-XL/BCL-2 inhibitor developed by AstraZeneca, AZD4320^13,27–29^ was designed for once weekly intravenous delivery rather than daily oral administration. Whilst thrombocytopenia is still observed, it is transient, and platelet levels recover within 48–72 h. However, more recent pre-clinical studies, have also revealed dose-limiting cardiotoxicity^30^. To overcome these issues as well as improve physicochemical limitations such as solubility, AstraZeneca recently reported a new BH3-mimetic where AZD4320 is conjugated *via* a hydrolytically labile linker to the clinically validated DEP^®^ dendrimer nanomedicine platform^30^. This novel molecule mitigates the cardiovascular effects observed with AZD4320, and has shown significant efficacy in vitro and in vivo in a range of hematological and solid cancers^30^. Accordingly, AZD0466 is now undergoing a clinical trial (NCT04214093) for hematological and advanced solid cancers.

In this study we show that AZD0466 has potent activity in MPM, with less thrombocytopenia compared to that observed with other BH3-mimetics targeting BCL-XL. It also requires significantly reduced dosing to achieve outcomes similar to what we have previously reported in pre-clinical models of MPM, underscoring its efficacy relative to first generation unconjugated BH3-mimetics.

## Materials and methods

### Drugs

AZD4320, AZD5991 and AZD0466 were provided by Astra Zeneca. Stock solutions of AZD4320 and AZD5991 for in vitro analysis were prepared in dimethyl sulfoxide (DMSO). Cisplatin was purchased from Selleckchem and prepared in dimethyl formamide (DMF) for in vitro analysis.

### Immunohistochemical analysis of tumor samples ex vivo

Immunohistochemical analysis for BCL-2 family protein expression was performed as described previously^26^ using antibodies from Cell Signaling Technology (USA) against BCL-XL (clone 54H6, cat# 2764), MCL-1 (clone D5V5L, cat# 39224), BAK (clone D4E4, cat# 12105), BAX (clone D2E11, cat#5023), BIM (clone C34C5, cat# 2933), BCL-2 (clone 124, cat# 15071) and cleaved Caspase-3 (clone Asp175, cat# 9661) or Ki67 (clone 30-9, cat# 790-4286) from Roche (USA). The intensity of the cytoplasmic immunostaining with the BCL-2 family protein antibodies was evaluated as: 0, no staining; 1, weak; 2, moderate; and 3, strong. The extent of tumor cell cytoplasmic immunostaining was evaluated as: 0, no immunostaining; 1, 1–5% positive; 2, 6–25% positive; 3, 26–50% positive; 4, 51–75% positive; and 5, 76–100% positive. A combined H-score was determined by multiplying the intensity and extent scores, giving a final value between 0–15. Quantification of cleaved Caspase-3 and Ki67 staining was performed through division of the number of positive cells by the number of all tumor cells per high power field (20x) and the result was given as the mean of five randomly evaluated regions for all tumor samples.

### Cell culture

Human MPM cell lines (MSTO-211H; biphasic and NCI-H28; epithelioid) were obtained from the American Type Culture Collection (ATCC, USA). All lines were confirmed to be mycoplasma negative based on in-house MycoAlert assays (Lonza). Cells were cultured in RPMI 1640 medium (cat#11875093, Gibco), supplemented with heat-inactivated 10% (v/v) fetal calf serum (PAA Laboratories, Australia), 1% (v/v) GlutaMAX Supplement 200 mM l-alanyl-l-glutamine dipeptide in 0.85% (w/v) NaCl (Gibco) and 100 U/ml penicillin, 100 mg/ml streptomycin (Gibco) and maintained at 37 °C with 5% CO_2_.

### CellTiter-Glo luminescent viability assay

Cells (1000 per well) were seeded into 96-well white plates and 4 h later treated with serial dilutions of vehicle (DMSO or DMF), drugs alone, or combinations thereof. Cell viability was assessed after 72 h treatment using the CellTiter-Glo 2.0 assay (cat# G9243, Promega, Australia) following the manufacturer’s instructions. Luminescence was measured on an Ensight Multimode plate reader (Perkin Elmer). The results were normalized to the viability of cells treated with the highest % (v/v) of vehicle. The EC_50_ values were calculated using non-linear regression algorithms using the Prism software (Graph Pad, version 6) from the combined data of at least three separate experiments. Synergy analysis was performed using Combenefit software with Bliss, Loewe and HSA models^31^.

### FACS-based apoptosis assay

Cells (30,000 per well) were seeded into 24-well plates, then 24 h later treated with vehicles, drugs, either alone or in combination. For pan-caspase inhibitor assays, Q-VD-OPh (cat# 03OPH10905, MP Biomedicals) was added (final concentration 25 µM) to respective wells. Live and dead cells were harvested and pelleted by centrifugation and incubated with Annexin V-APC (cat# 550475, BD Biosciences) and propidium iodide (cat# p4864, Sigma Aldrich) in Annexin V binding buffer (cat# 556454, BD Biosciences).

FACS analysis was performed on a BD FACSCanto II flow cytometer (BD Biosciences, USA). Data was analyzed using FlowJo Software Version 10 (FlowJo-LLC, USA) with the number of viable cells (Annexin V negative / propidium iodide negative) normalized relative to the number of viable cells cultured in the vehicle control. GraphPad Software was used for statistical analysis.

### Quantitative real-time polymerase chain reaction

Total RNA was extracted from freshly frozen tumor samples using the ReliaPrep RNA Cell Miniprep System (Promega, USA) and reverse-transcribed using random hexamers and the Transcriptor High Fidelity cDNA synthesis kit (Roche, Germany) as per the manufacturer’s instructions. Quantitative RT-PCR was performed using Power SYBR Green PCR Master Mix (Roche, USA) with previously-published primer sets^32^ on a ViiA^TM^ 7 Real-Time PCR System (Applied Biosystems, USA).

### Mouse xenograft experiments

NOD-*scid* 6–8 week old female mice were purchased from the Animal Resource Centre (Perth, Western Australia) and maintained at the Bio-Resources Facility at Austin Health. All animal experiments were approved by the Austin Health Animal Ethics Committee (A2017_05465/ A2018_05584) and carried out in accordance with the guidelines of Austin Health/University of Melbourne and conformed to the National Health and Medical Research Council’s code of practice for the care and use of animals for scientific purposes.

MSTO-211H cells (4 × 10^6^) in Matrigel (BD Biosciences, Australia) were injected subcutaneously into the right flank of mice. When tumors reached 150–200 mm^3^, the mice were randomized into different treatment groups to ensure consistent average tumor size across all study arms (*n* = 6–10 per group based on previous outcomes of drug treatment studies using MPM xenografts) that received either PBS (intra-peritoneal injection), AZD0466 vehicle (citrate/phosphate buffer, pH 5.0; intravenous injection), Cisplatin (4 mg/kg; intra-peritoneal injection), AZD0466 (100 mg/kg; intravenous injection), Cisplatin *plus* AZD0466 vehicle (4 mg/kg; intra-peritoneal and intravenous injections respectively) or Cisplatin *plus* AZD0466 (4 mg/kg and 100 mg/kg; intra-peritoneal and intravenous injections respectively). Cisplatin and associated vehicle controls were administered on Day 1, whilst AZD0466 (and associated vehicle controls) were administered on Days 1 and 8.

Tumors were measured using callipers three times a week by a blinded investigator (SA) and three mice per treatment group were culled by CO_2_ asphyxiation at the end of the study treatment phase (19 days from start of treatment, i.e., 10 days after final intravenous dosing). The remaining mice were culled either at ethical endpoint when tumors reached 1000 mm^3^ or if there was evidence of toxicity (weight loss >15%). Although early termination during the study treatment phase was a pre-defined censoring event, no mice in the treatment cohorts lost weight beyond that approved on the ethics protocol. Blood was collected by retro-orbital bleed 48 h after dosing with AZD0466, 10 days after start of treatment in microcuvette tubes (Sarstedt). Full blood examination was carried out on an Advia 2120 blood analysis machine (Siemens) according to the manufacturer’s instructions. Tumors were excised, measured and weighed, and subsequently paraffin-embedded for IHC analysis or snap frozen for cell lysate preparation for qRT-PCR.

## Results

### AZD4320 is effective on MPM cell lines and synergizes with an MCL-1 inhibitor

To investigate the potential efficacy of AZD0466 in MPM, we initially used CellTiter-Glo viability assays to test the effect of the active moiety, AZD4320. AZD4320 provides similar outcomes on cell lines to AZD0466, though with kinetics more suitable for testing in in vitro assays due to the slower release of AZD4320 from the AZD0466 dendrimer. Here, we used identical conditions (e.g. cell numbers, volumes, incubation times) to those we recently reported for other BH3-mimetic compounds we have examined in MPM to enable meaningful comparisons between them^9^. We also used two cell lines, NCI-H28 representative of the most common MPM histological subtype (epithelioid), and MSTO-211H (biphasic), a line that we have previously employed in in vivo studies^9^.

**Fig. 1:**
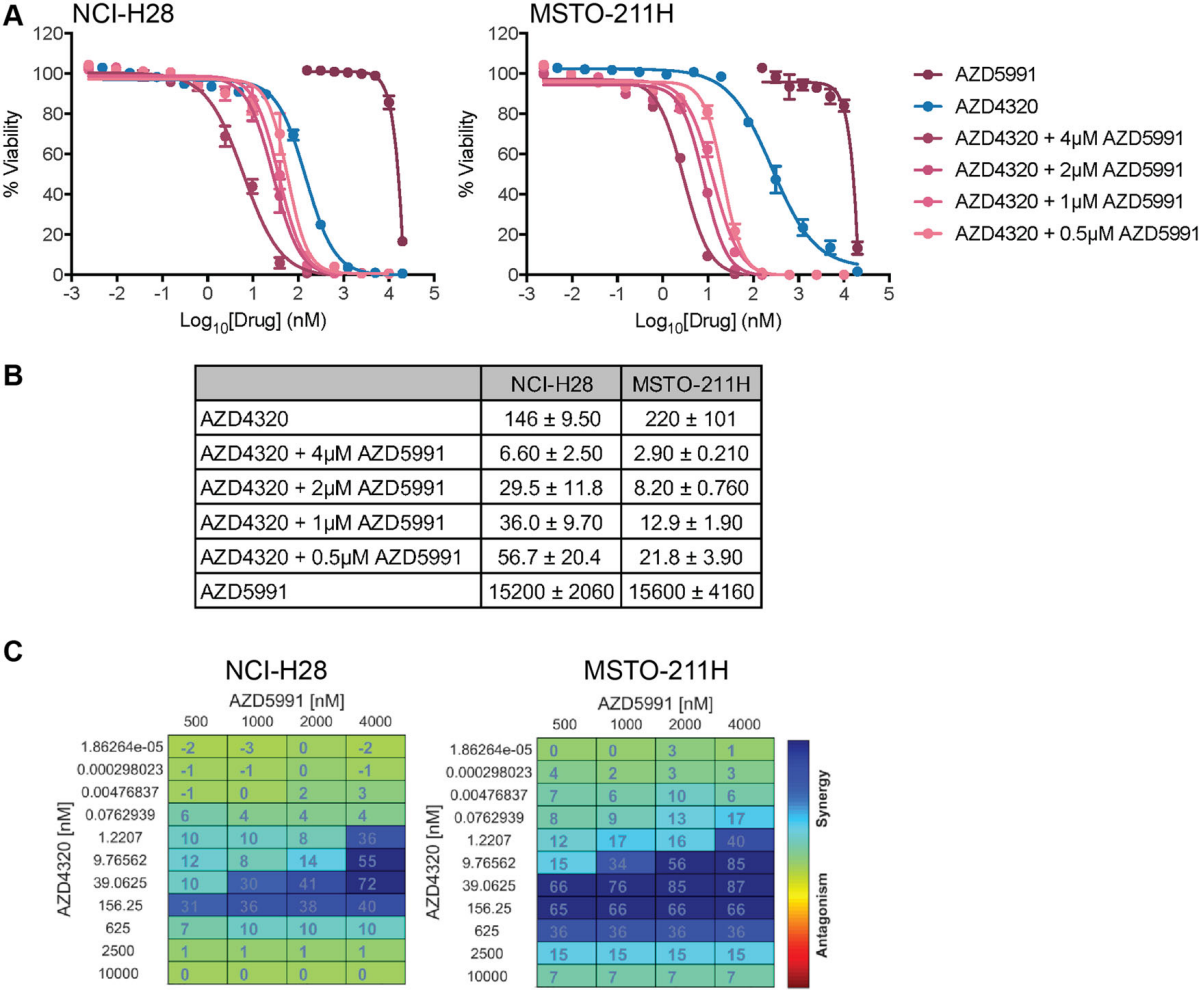
AZD4320 inhibits MPM cell viability in vitro. **A** AZD4320 alone inhibits MPM cell line viability, and this effect is enhanced by co-treatment with AZD5991. Cell viability was determined using CellTiter-Glo assays after 72 h treatment. Data represent mean ± SEM (*n* = 3). **B** Summary of EC_50_ values for treatments in **A**. **C** Synergy analysis of BH3-mimetic drug combinations was performed with Combenefit software using the BLISS synergy model. Strong synergy was observed across a range of drug concentrations.

Single agent activity of AZD4320 was observed with EC_50_ values (150–220 nM, Fig. 1A, B) similar to what we previously observed for Navitoclax (310–830 nM) on the same cell lines, though it was not as potent as A-1331852, a more specific BCL-XL inhibitor (EC50 ~4–11 nM)^9^. As we previously showed that MCL-1 is a significant barrier to maximum efficacy with A-1331852 and Navitoclax, we also combined AZD4320 with AZD5991, a potent MCL-1 inhibitor from AstraZeneca^19^. These data mirrored what we saw previously with combinations of A-1331852 or Navitoclax with another MCL-1 inhibitor (S63845), where significant improvements in EC_50_ values were achieved with increasing doses of the MCL-1 inhibitor (Fig. 1A, B). AZD5991 alone was inactive (EC_50_ > 15 μM), as seen with S63845^9^, though when combined with AZD4320, EC_50_ values in the range of 3–55 nM were observed (depending on the AZD5991 concentration). This increased activity was synergistic across a range of drug dose combinations, as assessed using BLISS analysis (Fig. 1C).

Combined, these data demonstrate that AZD4320 is as potent as previously investigated BH3-mimetic drugs that we have shown to have in vivo activity on MPM, and are consistent with our previous findings that co-targeting of BCL-XL and MCL-1 synergistically induces MPM cell killing.

### AZD4320 reduces MPM cell viability via apoptosis induction

**Fig. 2:**
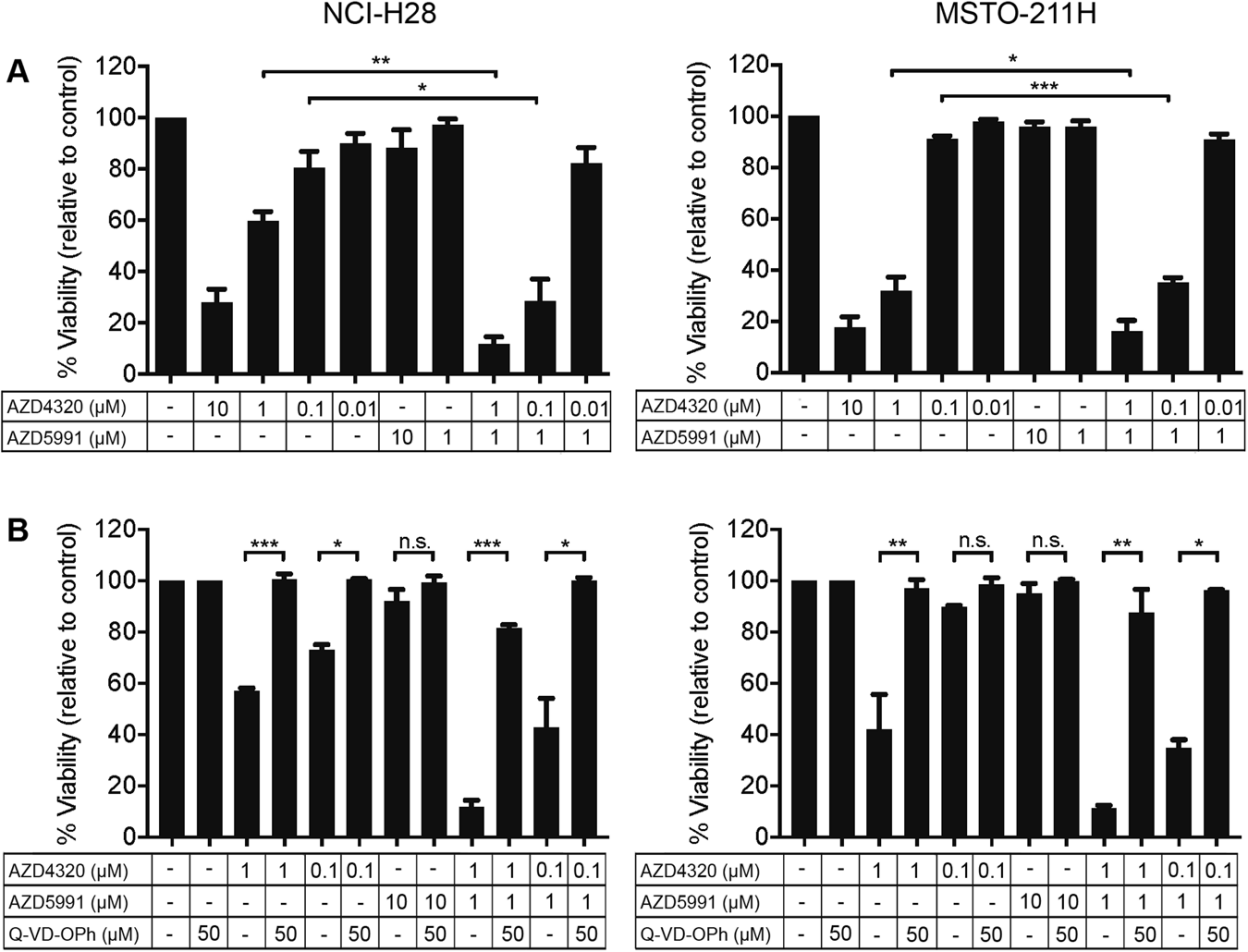
AZD4320 induces apoptosis in MPM cell lines. **A** Indicated cell lines were treated with AZD4320, AZD5991, or both for 72 h, and apoptosis induction monitored by FACS using Annexin V / propidium iodide staining. **B** Apoptosis induction was confirmed by treating cells with drugs as single agents and in combination in the presence of the pan-caspase inhibitor Q-VD-OPh. Data represent mean ± SEM (*n* = 3) *p ≤ 0.05, **p ≤ 0.01 and ***p ≤ 0.001 (unpaired Students *t*-test).

The CellTiter-Glo viability assays measure cellular ATP levels, hence, changes could reflect effects on either cell proliferation or apoptosis induction (or both). To confirm that AZD4320 was inducing apoptosis as expected, flow cytometry assays were performed using well-established markers of apoptosis (Annexin V and propidium iodide staining) and showed dose-dependent apoptotic cell killing with AZD4320. Moreover, when doses of AZD4320 that provided 65% or less cell killing (i.e., at 1 μM or less) were combined with 1 μM AZD5991 (where essentially no killing was observed), the effect was enhanced (Fig. 2A), closely reflecting the outcomes of the CellTiter-Glo assays. To confirm apoptosis was the dominant mechanism-of-action, AZD4320 was tested alone or in combination with AZD5991 in the presence of the pan-caspase inhibitor, Q-VD-OPh. Consistent with the loss of viability being through apoptosis, Q-VD-OPh rescued the effect of AZD4320 on its own and in combination with AZD5991 (Fig. 2B). Hence, these data confirm that the mechanism-of-action of AZD4320 (and AZD5991) is via apoptosis induction, in line with their capacity to engage their pro-survival protein targets with high affinity.

### AZD4320 enhances the effect of Cisplatin in vitro

**Fig. 3:**
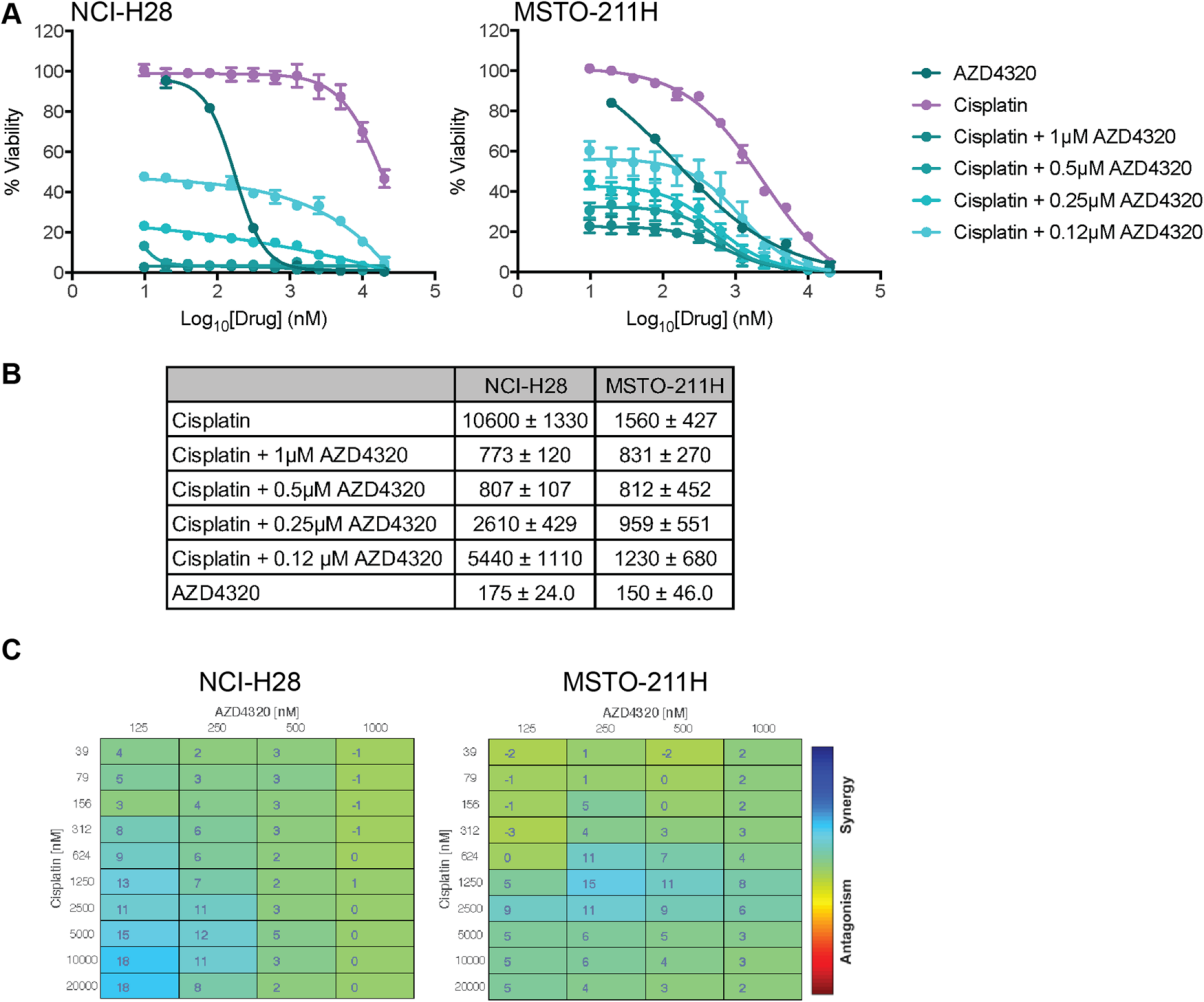
AZD4320 enhances MPM cell killing by Cisplatin. **A** Cisplatin alone is less effective than AZD4320, however, the combination increases cell killing. Cell viability was determined using CellTiter-Glo assays after 72 h treatment. Data represent mean ± SEM (*n* = 3). **B** Summary of EC_50_ values for treatments in **A**. **C** Synergy analysis of combinations in **A** performed with Combenefit software using the BLISS synergy model.

Standard drug development protocols necessitate that any new agents are combined with standard of care treatment regimens, which for mesothelioma currently includes Cisplatin. As our previous studies showed that BCL-XL inhibition (using ABT-263 or A-1331852) could enhance the effects of Cisplatin, both in vitro and in vivo^9^, it was of interest to determine whether AZD4320 could act similarly. Accordingly, we also examined the effect of AZD4320 in combination with Cisplatin on cell viability. As observed with other BH3-mimetics capable of inhibiting BCL-XL, some enhanced activity was observed when AZD4320 was combined with Cisplatin, though this was cell-line dependent with NCI-H28 cells showing the greater relative increase in response with the combinations (14-fold versus 2-fold when Cisplatin was titrated in the presence of 1 μM AZD4320). However, it should be noted that this cell line was significantly more resistant to Cisplatin alone (~7-fold), compared to MSTO-211H cells (Fig. 3A, B). For both cell lines, these responses were weakly synergistic (Fig. 3C). Hence, AZD4320 displays similar behavior to other BCL-XL antagonists in its capacity to act in co-operation with Cisplatin to kill MPM tumor cells.

### AZD0466 is efficacious on MPM xenografts and its activity is enhanced by Cisplatin co-treatment

As AZD4320 can potently kill MPM cell lines in vitro, we next assessed whether the dendrimer incorporating this drug, AZD0466, could exert similar activity in vivo. Here, we used MSTO-211H xenografts to enable direct comparison with other BH3-mimetics we have previously tested in vivo using this cell line^9^, and because we were unable to establish tumors in mice for the NCI-H28 cell line. Pilot dose escalation studies in non-tumor bearing mice using AZD0466 at 25 mg/kg–100 mg/kg (once weekly for two weeks), together with a single dose of Cisplatin (4 mg/kg) demonstrated that the highest dose was well tolerated based on the minimal weight loss (<15%) observed over the treatment period. Hence, this dosing schedule was used in our xenograft studies.

**Fig. 4:**
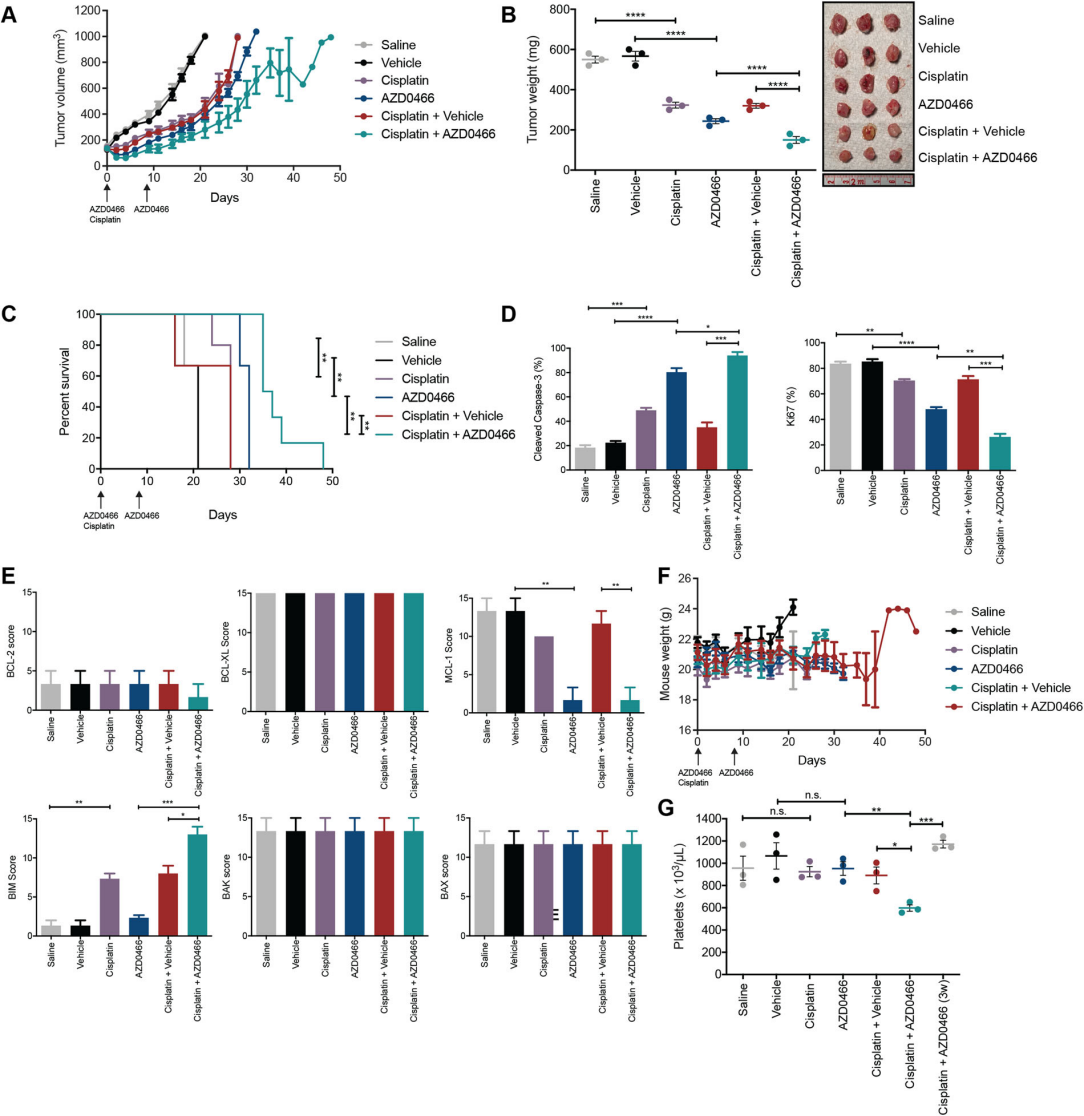
AZD0466 treatment results in tumor growth control of MPM xenografts. **A** MSTO-211H xenograft tumor volumes measured during and following treatment with AZD0466 (100 mg/kg, days 1 and 8, IV) and Cisplatin (4 mg/kg, day 1 IP), the combination, or appropriate controls. **B** Tumor masses at Day 19 from start of treatment. Each point is the mass of an individual tumor (photographed) with the bar indicating the mean ± SEM (*n* = 3) and significance determined by Student’s *t*-test (unpaired). **C** Kaplan–Meier survival curves of mice treated with AZD0466, Cisplatin and combinations of both, with relevant vehicle controls. Survival endpoint was when tumors reached 1000mm^3^ as dictated by the ethics approval associated with this experiment. Significance determined by Log-rank (Mantel–Cox test). **D** Immunohistochemistry analysis of tumors for cleaved Caspase-3 (CC3) and Ki67. Values represent the mean % postively stained cells for each antibody in five different fields of view. Data are mean ± SEM (*n* = 3), significance determined by Student’s *t*-test (unpaired). **E** Effect of AZD0466 and Cisplatin treatment on indicated BCL-2 family protein expression determined by immunohistochemistry (H-scores) on tumors harvested at Day 19 for indicated BCL-2 family members. Sections were scored for staining by each antibody in 5 different fields of view. Data are mean ± SEM (*n* = 3 tumors per group), significance determined by Student’s *t*-test (unpaired). **F** Body weights of mice were measured during and after the treatment period. Data represent mean ± SEM (n = 6–10). **G** Platelet counts (units per μL blood) determined 48 h post AZD0466 or vehicle dosing on Day 10 in all treatment groups and in Cisplatin *plus* AZD0466 treated mice 3 weeks (3w) post start of the treatment phase. Data is mean ± SEM (*n* = 3 mice per group), significance determined by Student’s *t*-test (unpaired).

Similar to what we observed with A-1331852 and Navitoclax^9^, AZD0466 alone was able to inhibit tumor growth (volume and mass) and extend median mouse survival (i.e., time to ethical endpoint tumor size of 1000mm^3^) to 32 days compared to Cisplatin alone (28 days) or vehicle controls (21 days, *p* ≤ 0.01) (Fig. 4A–C). The combination of AZD0466 with Cisplatin further increased median survival to 36 days. As expected, tumors resected from a subset of mice 10 days following the final dose of intravenous treatment displayed evidence of increased apoptosis and decreased proliferation, as determined by immunohistochemical analysis of cleaved Caspase-3 and Ki67 respectively (Fig. 4D). These changes were more pronounced in the AZD0466-treated tumors *versus* those treated with Cisplatin alone, and most pronounced in the combination treatment group. Immunohistochemical (Fig. 4E) and quantitative RT-PCR (Fig. S1) analysis of BCL-2 family protein and mRNA levels in tumors following treatment showed that mRNA and protein expression of MCL-1 was decreased following AZD0466 treatment, while BIM mRNA and protein expression was increased following Cisplatin treatment.

As observed in our pilot study, AZD0466 treatment was tolerated over the treatment period with no overt sign of clinical toxicity in any treatment group or weight loss (<15%) compared to the control groups (Fig. 4F). Blood samples were analyzed 48 h post AZD0466 treatment as our ethics approval for these experiments proscribed sampling within 2 days of intravenous drug injection. Notably, there was no evidence of thrombocytopenia in the AZD0466 treatment group at this time point, and whilst the platelet count was reduced in the AZD0466 plus Cisplatin treatment group, the degree of thrombocytopenia was relatively mild (i.e. mean platelet counts of 597/μL in the treatment groups *versus* 1066/μL in controls) (Fig. 4G). These platelet levels rebounded one week after treatment cessation. Hence, AZD0466 has significant impact on MPM tumor growth, and this outcome is enhanced by co-treatment with Cisplatin, with minimal overall effect on platelet levels.

## Discussion

In this study we have shown that AZD0466, a novel dendrimer nanomedicine based on the dual BCL-XL/BCL-2 inhibitor AZD4320^13,27,28^, is as efficacious as Cisplatin in controlling tumor growth and overall mouse survival (as assessed by time to ethical endpoint), and combinations of these drugs leads to further increased benefit. This level of disease control mirrors what we observed previously with other well-characterized inhibitors that can target BCL-XL (i.e., A-1331852 and Navitoclax)^9^.

Thrombocytopaenia is a known consequence of BCL-XL inhibition due to the absolute dependence of platelets on this pro-survival protein for their survival, and has hindered the translation of BH3-mimetics targeting BCL-XL to the clinic. One of the key potential benefits of AZD0466 over these other inhibitors is that the level of thrombocytopenia associated with treatment appears to be reduced. Indeed, there was no detectable change in mean platelet levels 48 h after drug administration. One caveat here is that our ethics approval for this experiment precluded blood sampling within 48 h of intravenous drug delivery, hence, a direct comparison of this on-target toxicity associated with A-1331852 / Navitoclax treatment was not possible as analysis with those drugs was performed 24 h after oral drug delivery. However, as AZD0466 was only administered once weekly, rather than daily for 5 consecutive days (followed by a two day break) as was performed for A-1331852 / Navitoclax, the overall degree of thrombocytopenia with AZD0466 is inevitably significantly reduced as compared to those other drugs where platelets are depleted by >90% within 24 h of administration (which occurs daily). As reported by AstraZeneca, platelet cell numbers decreased within 24 h of AZD0466 administration, though (like with AZ4320), this is only transient and levels recover within 48–72 h, as we observed^30^. Hence, there is the potential for a greater therapeutic margin with respect to thrombocytopenia with AZD0466 compared with Navitoclax.

The fact that comparable efficacy was observed with two single doses of AZD0466 as compared with 14 days of treatment for ABT-263 and A-1331852 speaks to the advantages derived from using the dendrimer platform where the extended (~25 h) drug release half-life mediated *via* the hydrolytically cleaved linkers dampens the plasma C_max_ and enhances delivery to, and retention within the tumor to increase efficacy, whilst reducing the non-clinical cardiovascular effects observed with AZD4320 in rats^30^. Indeed, we observed no evidence of cardiotoxicity in our studies.

AZD0466 is currently being tested in a phase I first-in-human clinical trial which includes patients with solid and hematological tumors (NCT04214093). This study will provide important insight into the therapeutic window of this agent. Similar to our study (and the pivotal pre-clinical study^30^), AZD0466 is being dosed weekly in the phase I trial. Potentially, if a safe therapeutic window can be established, co-dosing of AZD0466 with an MCL-1 inhibitor could be feasible, and presents an attractive strategy, especially given that we and others, have shown that optimal tumor cell killing can be achieved with BCL-XL and MCL-1 co-inhibition^33–35^. To date, this has not been possible due to the acute hepatotoxicity that is observed when current BCL-XL inhibitors are co-dosed with MCL-1 inhibitors in mice^34^.

In summary, our data with AZD0466 indicates that novel nanomedicine delivery of BH3-mimetics can provide similar, if not increased efficacy in MPM, compared to currently available “unconjugated” BH3-mimetics, and can potentially overcome the major single-agent toxicity (i.e., thrombocytopenia) associated with BH3-mimetics targeting BCL-XL in general. Whether similar efficacy will be observed in other solid tumor types is currently unknown, however, the phase I trial currently underway with AZD0466 should provide some insights into this in the near future. Regardless, given the limited treatment options currently available for MPM patients, these promising initial data on AZD0466 in MPM suggests its future clinical evaluation in this disease should be considered.

## Supplementary information

Supplementary Figure Legends

Supplementary Figure 1
