# Supplementary material for: A novel BH3-mimetic, AZD0466, targeting BCL-XL and BCL-2 is effective in pre-clinical models of malignant pleural mesothelioma
Source: Cell Death Discov. 2021 May 28;7:122. doi: 10.1038/s41420-021-00505-0 (PMC8163735; doi:10.1038/s41420-021-00505-0)
Supplement: Supplementary file 1 — Supplementary Figure Legends [file 41420_2021_505_MOESM1_ESM.docx]

**Supplementary Information**

**Supplementary Figure Legend**

**Figure S1:** Expression of mRNA for BCL-2 family members in tumors harvested at Day 19 of the experiment determined by qRT-PCR. Data are mean ± SEM (n=3 tumors per group) and represented as a heatmap in the lower panel.
