# Supplementary figures and images for: A novel BH3-mimetic, AZD0466, targeting BCL-XL and BCL-2 is effective in pre-clinical models of malignant pleural mesothelioma
Source: Cell Death Discov. 2021 May 28;7:122. doi: 10.1038/s41420-021-00505-0 (PMC8163735; doi:10.1038/s41420-021-00505-0)

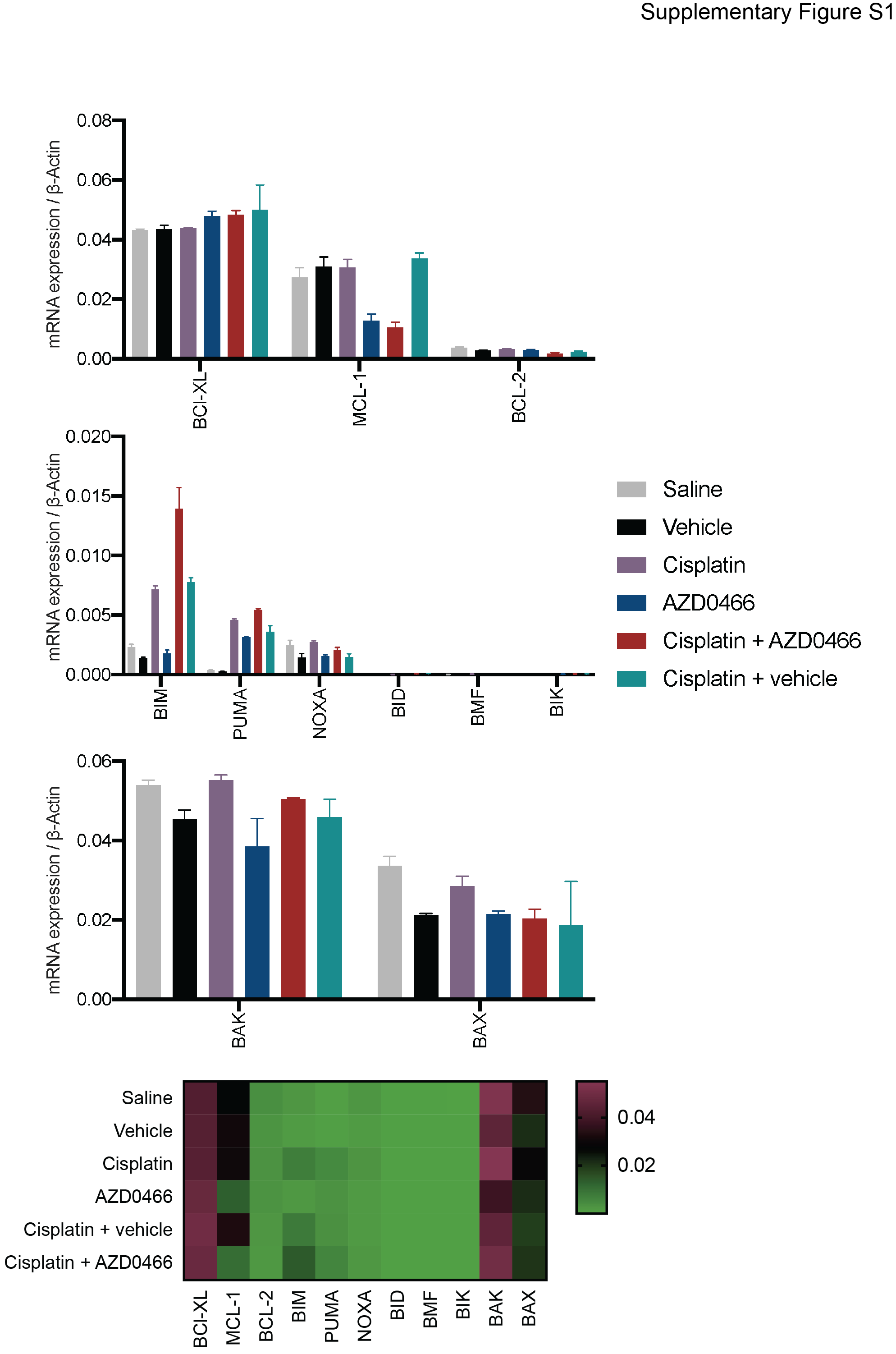

Supplement: Supplementary file 2 — Supplementary Figure 1 [file 41420_2021_505_MOESM2_ESM.png]
